# The genetic polymorphisms of HER-2 and the risk of lung cancer in a Korean population

**DOI:** 10.1186/1471-2407-8-359

**Published:** 2008-12-04

**Authors:** Uk Hyun Jo, Sle Gi Lo Han, Jae Hong Seo, Kyong Hwa Park, Jae Won Lee, Hyo Jung Lee, Jeong Seon Ryu, Yeul Hong Kim

**Affiliations:** 1 Department of Internal Medicine and Division of Brain Korea 21 Project for Biomedical Science, Korea University Anam Hospital, Korea University College of Medicine, Seoul, Korea; 2 Genomic Research Center for Lung and Breast/Ovarian Cancers, Korea University Anam Hospital, Korea University College of Medicine Seoul, Korea; 3 Division of Oncology/Hematology, Department of Internal Medicine, Korea University Anam Hospital, Korea University College of Medicine, 126-1, 5 ga, Anam-dong, Seongbuk-gu, Seoul, 136-705, Korea; 4 Department of Statistics, College of Political Science and Economics, Korea University, Seoul, Korea; 5 Department of Internal Medicine, Inha University College of Medicine, 3 ga Shinheung-dong, Incheon-si, 400-711, Korea

## Abstract

**Background:**

Human Epidermal Growth Factor Receptor 2 (HER-2; also known as erbB-2 or neu), a proto-oncogene of the receptor tyrosine kinase superfamily, has been associated with carcinogenesis and prognosis of human cancers, acting as a binding partner of other epidermal growth factor receptor (EGFR) family in the activation of EGFR signaling. Amplification of the HER-2 gene has been reported in lung cancer, where it has been associated with poor prognosis. In this study, we investigated whether the four polymorphisms (-3444C>T, -1985 G>T, I655A A>G and P1170A C>G) of the HER-2 gene are associated with the risk of lung cancer in Korean populations.

**Methods:**

The frequencies of 4 polymorphisms of the HER-2 gene were examined by the polymerase chain reaction-restriction fragment length polymorphism or the single-nucleotide polymorphism-identification technology assay in the 407 lung cancer patients and 407 healthy controls.

**Results:**

The frequencies of the 4 polymorphisms were not significantly different between patient and control groups in overall subjects. However, in the subgroup analysis, the 3 single nucleotide polymorphisms (-3444C>T, -1985G>T and P1170A C>G) showed statistically significant differences in the subgroups of females, non-smokers, and non-drinkers (*p *< 0.05). Additionally, we found the association between the risk of lung cancer and the polymorphisms of HER-2 gene in non-smoker subgroups with adenocarcinoma (*p *< 0.05).

**Conclusion:**

Our results suggest that the polymorphisms of the HER-2 gene are associated with an increased susceptibility to lung cancer in females, non-smokers and non-drinkers subgroups in the Korean population.

## Background

Lung cancer is the worldwide leading cause of cancer-related death [1]. During the past decades, the rate of incidence and mortality of lung cancer in Korea have been increasing significantly and constantly[2]. Although lung cancer has been considered as a disease caused by smoking and environmental/occupational exposure, previous studies suggest that genetic factors may also contribute to the risk of lung cancer [3].

Single nucleotide polymorphisms (SNPs) are the most common form of human genetic variation, and they may contribute to an individual's susceptibility to cancer [4,5]. Many previous studies have demonstrated that some polymorphisms of certain genes are associated with the risk of lung cancer, affecting either the gene expression or activities of enzymes [6-8].

The HER-2 (also known as erbB-2 or neu and a member of the epidermal growth factor receptor family), proto-oncogene is located at chromosome 17q21 and encodes a transmembrane glycoprotein (p185) with tyrosine kinase activity [9,10]. Somatic mutations in the HER-2 gene have not been observed in humans, however amplification and overexpression of the HER-2 protein occur in lung cancer and contribute to poor prognosis [11-13]. Recently, several polymorphisms of the HER-2 gene have been deposited in public databases . Previous studies have suggested functional implications of the polymorphisms by showing that the polymorphisms of the HER-2 gene might result in increased autophosporylation and tyrosine kinase activation [14,15]. It is possible that the polymorphisms are associated with lung cancer, modulating the susceptibility to disease.

To test this hypothesis, we performed a case-control study to evaluate the association between the polymorphisms of the HER-2 gene and the risk of lung cancer in the Korean population.

## Methods

### Study subjects

All 814 study subjects were recruited between August 2001 and June 2005, including 407 lung cancer patients recruited from the patient pool at the Genomic Research Center for Lung and Breast/Ovarian Cancers (Seoul, Korea) and Inha University Medical Center (Incheon, Korea). The histological classification and staging of all lung cancer cases was performed by pathological evaluation at the time of diagnosis. 407 control subjects randomly selected from a pool of healthy volunteers who had visited the Cardiovascular Genome Center (Seoul, Korea), Genome Research Center for Allergy and Respiratory Disease (Seoul, Korea), and Keimyung University Dongsan Medical Center (Daegu, Korea). Detailed information on diet, smoking status, drinking status, lifestyle, and medical history were collected by a trained interviewer using a structured questionnaire. This study was also approved by the institutional review board of participating institutes, and a written informed consent was obtained from each participant.

### Selection of the polymorphisms for the HER-2 gene

**Figure 1:**
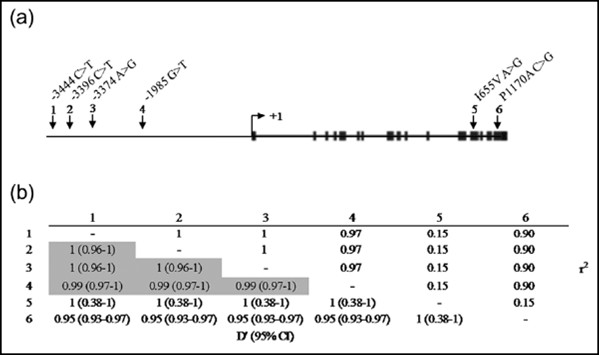
**Distribution of HER-2 polymorphism**. (a) The locations of the six SNPs used in the HER-2 genotyping analysis [vertical arrows (numbered 1 to 6 from 5' to 3'): 1, rs2643194; 2, rs2517951; 3, rs2643195; 4, rs2934971; 5, rs1801200; 6, rs1058808]. The translation start site is marked with right-angled arrow at +1. Exons are dipicted as small blocks. (b) The pairwise measure of linkage disequilibrium (LD) between the six SNPs in (a) with D' statistics (values in the bottom left area of the table). The pairs exhibiting a strong linkage disequilibrium pattern are shaded. Especially, the three SNPs (numbered in 1, 2, 3) are in completed LD. r2 values are shown on the top right area of the table.

6 SNPs of HER-2 gene were chosen in the promoter regions (-3444 C>T, -3396 C>T, -3374 A>G, -1985 G>T) and 2 SNPs in the coding regions (I655A A>G and P1170A C>G) (Fig. 1a). -3444 C>T, -3396 C>T, -3374 A>G, and -1985 G>T polymorphism was found by the direct-sequencing for the region spanning ~4kb upstream from the translation initiation site (NM_004448) of HER-2 gene in a small set of 24 lung cancer cases. I655A A>G and P1170A C>G polymorphisms were selected by reviewing the previous reports in the Korean population [16,17].

### Genotyping

Genomic DNA was prepared from 814 peripheral blood samples using Puregene blood DNA kit (Gentra, Minneapolis, MN). Polymerase chain reaction-restriction fragment length polymorphism (PCR-RFLP) and single-nucleotide polymorphism-identification technology (SNP-IT) assay were performed for genotyping of HER-2 polymorphisms.

Genotyping of -1985 G>T and I655V A>G SNPs was carried out by PCR-RFLP. The PCR primer sequences for -1985 SNP were: forward, 5'-ACC CCA GCA TAG TAT GTC AGA TG-3', and reverse, 5'-ATC CTA GGG AGT TGA GAA ACA GG-3'. The PCR products were digested by *Xmn I *and resolved by gel electrophoresis. The I655V SNP was analyzed as described previously [18].

-3444 C>T and P1170 C>G SNPs genotyping was performed by SNP-IT assay using the SNP stream 25 K System (Orchid Biosciences, Princeton, NJ). Briefly, the genomic DNA region spanning the polymorphic site was PCR-amplified using one phosphothiolated primer and one regular PCR primers. The amplified PCR products were then digested with exonuclease. The phosphothioated strand of the PCR product was protected from exonuclease digestion, generating a single-stranded PCR template. The single-stranded PCR template was overlaid on a 384-well plate that contained a covalently attached SNP-IT extension primer which was designed to hybridize immediately adjacent to the polymorphic site. The SNP-IT primer was extended for a single base with DNA polymerase and a mixture of appropriate dideoxynucleotide tagged with either fluorescein isothiocyanate (FITC) or biotin. The identity of the incorporated nucleotide was determined by serial colorimetric reactions with anti-FITC-alkaline phosphatase and streptavidin-horse radish peroxidase, resulting blue and/or yellow color developments were analyzed with an ELISA reader and the final genotyping (allele) calls were made with the QCReview program.

### Statistical analysis

Genotype frequencies and departures of genotype distributions from the Hardy-Weinberg equilibrium for each SNP were analyzed using the chi-square test or Fisher's exact test. Pairwise LD for calculating D' and r^2 ^was evaluated as described previously [19]. Linkage disequilibrium (LD) values were estimated using the Haploview version 3.32 . A pair of SNPs is defined to have "strong linkage disequilibrium" if the one-sided upper 95% confidence interval (95% CI) boundary on D' is >0.98 and the lower boundary is >0.7. Conversely, SNP pairs are said to have "strong evidence of historical recombination" if the upper CI boundary on D' is <0.9. Genotype-specific risks were estimated as odds ratios with associated 95% confidence intervals by unconditional logistic regression (SAS Institute, Cary, NC) and adjusted for age and gender. The p-value of < 0.05 was considered statistically significant. Unconditional logistic regression analysis was used to calculate odds ratios (ORs) and 95% confidence intervals (CIs) for overall lung cancer, with adjustment gender and age. In addition to the overall association analysis, we performed a stratified analysis by age (median age, = 60 years/>60 years), gender, smoking status, drinking status and tumor histology to further explore the association between HER-2 genotypes and the risk of lung cancer in each stratum. The ORs and 95% CIs in the stratification analyses were calculated using unconditional logistic regression analysis, with adjustment for gender and age, when appropriate.

## Results

We investigated the relationship between polymorphisms of HER-2 and the risk of lung cancer in a case-control study of 814 age-gender matched case and control subjects. The distributions of age, gender, smoking status and drinking status of every subject are summarized in Table 1. There were no differences in median age and gender between cases and controls. However, smoking status and drinking status were missing in some patients. These differences were controlled in the later logistic regression analysis with adjusted gender and age.

**Table 1:** Demographic characteristics of case-control study population

Characteristics	Controls	Cases	*p*-value
1. Age	*n *= 407	*n *= 407	
Median age	60.8	60.8	
Range	30 – 84	30 – 78	
2. Gender, *n*, (%)	*n *= 407, (%)	*n *= 407, (%)	
Male	302, (74.2)	303, (74.5)	0.94
Female	105, (25.8)	104, (25.6)	
3. Smoking status, *n*, (%)	*n *= 309, (%)	*n *= 396, (%)	
Non-smoker	162, (52.4)	108, (27.3)	<0.0001
Smoker	147, (47.6)	288, (72.7)	
4. Drinking status, *n*, (%)	*n *= 224, (%)	*n *= 310, (%)	
Non-drinker	78, (34.8)	118, (38.1)	0.44
Drinker	146, (65.2)	192, (61.9)	
5. Histological cell type, n, (%)		N = 388, (%)	
Adenocarcinoma		157, (40.5)	
Squamous cell		123, (31.7)	
Small cell		79, (20.4)	
Other carcinoma^**a**^		29, (7.5)	

^**a **^Large cell, mixed cell carcinomas or undifferentiated carcinoma

In this study, we have chosen 4 SNPs in the promoter regions (-3444 C>T, -3396 C>T, -3374 A>G, -1985 G>T) and 2 SNPs in the coding regions (I655A A>G and P1170A C>G) of the HER-2 gene (Figure 1a). Interestingly, the 3 SNPs (-3444 C>T, -3396 C>T and -3374 A>G) in the promoter regions showed a complete linkage disequilibrium (LD) (D' = 1)(Figure 1b). These results imply that the 3 polymorphisms are a SNP block. We representatively showed the data of -3444C>T for the 3 SNPs (-3444 C>T, -3396 C>T and -3374 A>G) in the following results. Therefore, we only performed genotyping for the 4 SNPs (-3444 C>T, -1985 G>T, I655A A>G and P1170A C>G). Therefore, we only performed genotyping for the 4 SNPs (-3444 C>T, -1985 G>T, I655A A>G and P1170A C>G) in all study subjects (407 case including the previous 24 case samples and 407 controls).

The frequencies of the 4 SNPs (-3444 C>T, -1985 G>T, I655A A>G and P1170A C>G) in the total lung cancer and healthy-control subjects are shown in Table 2. The distributions of the 4 SNPs in the cases and controls were in Hardy-Weinberg equilibrium. There were no significant differences between the 4 polymorphisms of the HER-2 and the risk of lung cancer in all subjects (Table 2). However, in the subgroup analysis, there were statistically significant differences between the genotype frequencies of the 3 SNPs (-3444 C>T, -1985 G>T and P1170A C>G) and the risk of lung cancer (Table 3, 4 and 5, Additional File 1). In females, -3444 TT and -1985 TT genotypes exhibited increased risk of lung cancer (Table 3). In non-smokers and non-drinkers, -3444 TT, -1985 TT and P1170A GG genotypes showed higher risk of lung cancer than other genotypes of the SNPs and the differences were more magnified after adjustment for age and gender (Table 4 and 5). Furthermore, we additionally performed the statistical analysis to demonstrate whether the polymorphisms are associated with tumor histology in the subgroups. We found that-3444 TT and -1985 TT genotypes exhibited the increased risk of lung cancer in non-smokers group with adenocarcinoma, adjusting with gender and age (Table 6). Hence, our results demonstrated that the 3 SNPs of HER-2 were associated with the risk of lung cancer in females, non-smokers, non-drinkers and tumor histology.

**Table 2:** Genotyping frequencies for HER-2 in cases and controls and their association with risk of overall lung cancer

	Genotype	Case (*n *= 407)	Control (*n *= 407)	asOR (95% CI)^**a**^	*p*-value
-3444 C>T	CC	141	140	1	
	CT	201	205	0.97 (0.72 – 1.32)	0.86
	TT	65	62	1.04 (0.68 – 1.58)	0.85
	CT+TT	266	267	0.99 (0.74 – 1.32)	0.94
	
	CC+CT	342	345	1	
	TT	65	62	1.06 (0.72 – 1.55)	0.77

-1985 G>T	GG	144	140	1	
	GT	196	205	0.93 (0.69 – 1.26)	0.64
	TT	67	62	1.05 (0.69 – 1.59)	0.82
	GT+TT	263	267	0.96 (0.72, 1.28)	0.77
	
	GG+GT	340	345	1	
	TT	67	62	1.10 (0.75-1.60)	0.63

I655V A>G	AA	304	300	1	
	AG	97	97	0.99 (0.71 – 1.36)	0.93
	GG	5	6	0.82 (0.25 – 2.72)	0.75
	AG+GG	102	103	0.98 (0.71 – 1.34)	0.88
	
	AA+AG	401	397	1	
	GG	5	6	0.83 (0.25 – 2.73)	0.75

P1170A C>G	CC	144	137	1	
	CG	199	206	0.92 (0.68 – 1.25)	0.59
	GG	64	64	0.95 (0.63 – 1.45)	0.82
	CG+GG	263	270	0.93 (0.69 – 1.24)	0.61
	
	CC+CG	343	343	1	
	GG	64	64	1.00 (0.69 – 1.46)	1

OR, odds ratio; CI, confidence interval.

^**a**^asOR are adjusted for age and gender.

**Table 3:** Comparative analysis between genotype frequencies and the risk of lung cancer in the subgroups of females

Female	Genotype	Cases (n = 104)	Controls (n = 105)	OR (95% CI)	*p*-value	asOR (95% CI)^**a**^	*p*-value
-3444 C>T	CC	35	39	1		1	
	CT	50	58	0.96 (0.53 – 1.74)	0.89	0.94 (0.51 – 1.72)	0.85
	TT	19	8	**2.65 (1.03 – 6.80)**	**0.04**	2.23 (0.85 – 5.84)	0.10
	CT+TT	69	66	1.17 (0.66 – 2.06)	0.60	1.11 (0.62 – 1.98)	0.73
	
	CC+CT	85	97	1		1	
	TT	19	8	**2.71 (1.13 – 6.51)**	**0.03**	2.31 (0.95 – 5.64)	0.07

-1985 G>T	GG	36	39	1		1	
	GT	49	57	0.93 (0.52 – 1.68)	0.81	0.93 (0.51 – 1.70)	0.81
	TT	19	9	2.29 (0.92 – 5.70)	0.08	1.93 (0.76 – 4.91)	0.17
	GT+TT	68	66	1.12 (0.63 – 1.97)	0.70	1.07 (0.60 – 1.92)	0.81
	
	GG+GT	85	96	1		1	
	TT	19	9	**2.38 (1.02 – 5.55)**	**0.04**	2.02 (0.85 – 4.78)	0.11

P1170A C>G	CC	37	37	1		1	
	CG	49	58	0.85 (0.47 – 1.53)	0.58	0.84 (0.46 – 1.55)	0.59
	GG	18	10	1.80 (0.73 – 4.42)	0.20	1.57 (0.63 – 3.91)	0.34
	CG+GG	67	68	0.99 (0.56 – 1.74)	0.96	0.96 (0.53 – 1.72)	0.88
	
	CC+CG	86	95	1		1	
	GG	18	10	1.99 (0.87 – 4.54)	0.10	1.73 (0.75 – 4.02)	0.20

OR, odds ratio; CI, confidence interval.

^**a**^asOR are adjusted for age.

**Table 4:** Comparative analysis between genotype frequencies and the risk of lung cancer in the subgroups of non-smokers

Non-smoker	Genotype	Cases (n = 108)	Controls (n = 162)	OR (95% CI)	*p*-value	asOR (95% CI)^**b**^	*p*-value
-3444 C>T	CC	36	61	1		1	
	CT	48	83	0.98 (0.57 – 1.69)	0.94	0.99 (0.56 – 1.74)	0.96
	TT	24	18	**2.26 (1.08 – 4.72)**	**0.03**	**2.67 (1.20 – 5.91)**	**0.02**
	CT+TT	72	101	1.21 (0.73 – 2.01)	0.47	1.25 (0.73 – 2.14)	0.41
	
	CC+CT	84	144	1		1	
	TT	24	18	**2.29 (1.17 – 4.46)**	**0.02**	**2.69 (1.30 – 5.56)**	**0.01**

-1985 G>T	GG	37	61	1		1	
	GT	47	82	0.94 (0.55 – 1.63)	0.84	0.97 (0.55 – 1.71)	0.91
	TT	24	19	**2.08 (1.01 – 4.31)**	**0.05**	**2.41 (1.10 – 5.27)**	**0.03**
	GT+TT	71	101	1.16 (0.70 – 1.93)	0.57	1.21 (0.71 – 2.07)	0.48
	
	GG+GT	84	143	1		1	
	TT	24	19	**2.15 (1.11 – 4.16)**	**0.02**	**2.46 (1.20 – 5.01)**	**0.01**

P1170A C>G	CC	37	58	1		1	
	CG	48	84	0.90 (0.52 – 1.54)	0.69	0.89 (0.50 – 1.57)	0.68
	GG	23	20	1.80 (0.87 – 3.73)	0.11	2.05 (0.94 – 4.47)	0.07
	CG+GG	71	104	1.07 (0.64 – 1.78)	0.79	1.09 (0.64 – 1.86)	0.76
	
	CC+CG	85	142	1		1	
	GG	23	20	**1.92 (1.00 – 3.71)**	**0.05**	**2.20 (1.08 – 4.47)**	**0.03**

OR, odds ratio; CI, confidence interval.

^**b**^asOR are adjusted for age and gender.

**Table 5:** Comparative analysis between genotype frequencies and the risk of lung cancer in the subgroups of non-drinkers

Non-drinker	Genotype	Cases (n = 118)	Controls (n = 78)	OR (95% CI)	*p*-value	asOR (95% CI)^**b**^	*p*-value
-3444 C>T	CC	38	23	1		1	
	CT	51	47	0.66 (0.34 – 1.26)	0.21	0.69 (0.35 – 1.35)	0.28
	TT	29	8	2.19 (0.86 – 5.61)	0.10	**2.85 (1.06 – 7.69)**	**0.04**
	CT+TT	80	55	0.88 (0.47 – 1.64)	0.69	0.96 (0.51 – 1.82)	0.90
	
	CC+CT	89	70	1		1	
	TT	29	8	**2.85 (1.23 – 6.62)**	**0.01**	**3.60 (1.47 – 8.84)**	**0.01**

-1985 G>T	GG	39	23	1		1	
	GT	50	47	0.63 (0.33 – 1.20)	0.16	0.67 (0.34 – 1.31)	0.24
	TT	29	8	2.14 (0.84 – 5.46)	0.11	**2.81 (1.04 – 7.56)**	**0.04**
	GT+TT	79	55	0.85 (0.46 – 1.57)	0.60	0.94 (0.50 – 1.78)	0.85
	
	GG+GT	89	70	1		1	
	TT	29	8	**2.85 (1.23 – 6.62)**	**0.01**	**3.60 (1.47 – 8.84)**	**0.01**

P1170A C>G	CC	40	22	1		1	
	CG	50	48	0.57 (0.30 – 1.10)	0.10	0.61 (0.31 – 1.21)	0.16
	GG	28	8	1.93 (0.75 – 4.94)	0.17	2.64 (0.97 – 7.18)	0.06
	CG+GG	78	56	0.77 (0.41 – 1.43)	0.40	0.86 (0.45 – 1.63)	0.64
	
	CC+CG	90	70	1		1	
	GG	28	8	**2.72 (1.17 – 6.34)**	**0.02**	**3.59 (1.46 – 8.88)**	**0.01**

OR, odds ratio; CI, confidence interval.

^**b**^asOR are adjusted for age and gender.

**Table 6:** Comparative analysis between genotype frequencies and the risk of lung cancer in non-smoker groups in adenocarcinoma-controls

Non-smoker	Genotype	Adenocarcinoma (n = 75)	Controls (n = 162)	OR (95% CI)	*p*-value	asOR (95% CI)^**b**^	*p*-value
-3444 C>T	CC	26	61	1		1	
	CT	33	83	0.93 (0.51 – 1.72)	0.82	0.91 (0.48 – 1.72)	0.77
	TT	16	18	2.09 (0.92 – 4.71)	0.08	2.37 (0.98 – 5.71)	0.06
	CT+TT	49	101	1.14 (0.64 – 2.02)	0.66	1.14 (0.63 – 2.06)	0.68
	
	CC+CT	59	144	1		1	
	TT	16	18	**2.17 (1.04 – 4.54)**	**0.04**	**2.49 (1.11 – 5.59)**	**0.03**

-1985 G>T	GG	27	61	1		1	
	GT	32	82	0.88 (0.48 – 1.62)	0.69	0.88 (0.47 – 1.66)	0.69
	TT	16	19	1.90 (0.85 – 4.25)	0.12	2.10 (0.88 – 5.00)	0.09
	GT+TT	48	101	1.07 (0.61 – 1.90)	0.81	1.09 (0.60 – 1.96)	0.79
	
	GG+GT	59	143	1		1	
	TT	16	19	**2.04 (0.98 – 4.24)**	**0.06**	**2.26 (1.03 – 5.00)**	**0.04**

P1170A C>G	CC	28	58	1		1	
	CG	32	84	0.79 (0.43 – 1.45)	0.44	0.75 (0.40 – 1.41)	0.37
	GG	15	20	1.56 (0.69 – 3.48)	0.28	1.64 (0.69 – 3.91)	0.26
	CG+GG	47	104	0.94 (0.53 – 1.65)	0.82	0.90 (0.50 – 1.64)	0.74
	
	CC+CG	60	142	1		1	
	GG	15	20	1.78 (0.86 – 3.70)	0.13	1.93 (0.87 – 4.27)	0.10

OR, odds ratio; CI, confidence interval.

^**b **^asOR are adjusted for age and gender.

## Discussion

In this study, we investigated whether the polymorphisms of the HER-2 gene were associated with the risk of lung cancer in the Korean population. Although the distribution of the polymorphisms of the HER-2 gene did not display statistically significant associations with all lung cancer and control subjects, the 3 polymorphisms (-3444 C>T, -1985 G>T and P1170A C>G) of HER-2 gene were found to be associated with the risk of lung cancer in female, non-smoker and non-drinker subgroups.

The previous studies on SNPs of the HER-2 gene have mainly been focused on breast cancer [14,18,20]. In the Korean population, Han et al. [16] showed that the polymorphisms of the HER-2 gene are associated with HER-2 protein expression and disease outcome in breast cancer. On the other hand, there are some reports to show that overexpression of HER-2 is an independent prognostic factor of survival and to be a combinatorial target in lung cancer, using an EGFR tyrosine kinase inhibitor together with HER-2 dimerization inhibitors [12,13,21]. Although cigarette smoking is the most important risk factor of lung cancer, previous studies have suggested differences in epidemiologic characteristics and environmental subtypes, suggesting an existence of non-tobacco related risk factors in the pathogenesis of lung cancer [22-24]. Alcohol consumption is an established risk factor for cancers of the oral cavity, pharynx, larynx, esophagus, and liver [25]. The associations between alcohol drinking and lung cancer risk have also been reported [26,27]. Moreover, somatic mutations of EGFR and HER-2 were preferentially identified in Oriental ethnicity compared with other ethnicities, female gender compared with male gender and never smokers compared with smokers, showing clinical responses to treatment [28-30]. Among the total 407 lung cancer patients, 89 patients were female and non-smoker in this study. Interestingly, about 72% (64/89) of non-smoker, female lung cancer patients were diagnosed as adenocarcinoma. We showed significant association between the polymorphisms and adenocarcinoma in the subgroups. Our results, therefore, suggest that the polymorphisms of the HER-2 gene might contribute to the susceptibility of female, non-smoker and non-drinker subgroups in the Korean population to lung cancer as EGFR mutations. However, the significant difference of the polymorphisms in females disappeared after adjustment for age. These discrepant results might be caused by the small number of young women in our study subjects [31]. Thus, our results need to be confirmed in future with a larger sample size of young women.

I655V A>G and P1170A C>G of HER-2 gene are located in the coding region that can affect protein structure and enzyme activity. In our results, I655V A>G was not associated with the risk of lung cancer in the Korean population, in consistance with previous study [16]. On the other hand, P1170A C>G was related to the risk of lung cancer in non-smokers and non-drinkers. These results suggest that P1170A C>G polymorphism may influence the activation of HER-2 gene, affecting the binding activity of specific effector proteins in lung cancer. Indeed, a recent review article describes several HER-2 binding partners [32], and a previous report also showed that the substitution of amino acid residue in the functional domain of the proteins can destabilize the protein-protein interactions [33]. Further studies are needed to clearly elucidate the relationship between the polymorphism and HER-2 activation.

To strengthen the significance of the present findings, some further studies have to be performed. First, ethnic difference of the polymorphisms of HER-2 gene should more clearly be established, because some polymorphisms of genes including HER-2 have different distribution in multiethnic population [34,35]. Second, the sample size of the subgroups of females, non-smokers and non-drinkers should be increased.

## Conclusion

Taken together, we conclude that the polymorphisms of HER-2 gene do not play strong roles in lung cancer susceptibility in the majority of the Korean population. However, we first demonstrated that the polymorphisms of HER-2 gene are associated with increased risk of lung cancer within the subgroups of females, non-smokers and non-drinkers in the Korean population. Our results can contribute to understanding susceptibility of specific subgroups in lung cancer.

## Competing interests

The authors declare that they have no competing interests.

## Authors' contributions

UHJ carried out all experiments and wrote this manuscript. SGLH performed a polymerase chain reaction-restriction fragment length polymorphism assay. JWL and HJL did statistical analysis. JHS, KHP and JSR selected cases, reviewed medical records and sample collection. YHK conceived of the study, and participated in its design and coordination. All authors read and approved the final manuscript.

## Pre-publication history

The pre-publication history for this paper can be accessed here:



## Supplementary Material

Additional File 1 Supplementary Tables, showing the comparative analysis between genotype frequencies and the risk of lung cancer in the subgroups of males (Table S1), smokers (Table S2) and drinkers (Table S3). Click here for file
