# Supplementary material for: The genetic polymorphisms of HER-2 and the risk of lung cancer in a Korean population
Source: BMC Cancer. 2008 Dec 4;8:359. doi: 10.1186/1471-2407-8-359 (PMC2661000; doi:10.1186/1471-2407-8-359)
Supplement: Additional File 1 — Supplementary Tables, showing the comparative analysis between genotype frequencies and the risk of lung cancer in the subgroups of males (Table S1), smokers (Table S2) and drinkers (Table S3). [file 1471-2407-8-359-S1.doc]

**Additional File 1, Supplementary Table S1- Comparative analysis between genotype frequencies and the risk of lung cancer in the subgroups of males**

| Males | Genotype | Cases (n=303) | Controls (n=302) | OR (95% CI) | *p*-value | asOR (95% CI) **^a^** | *p*-value |
| --- | --- | --- | --- | --- | --- | --- | --- |
| -3444 C>T | CC | 106 | 101 | 1 |  | 1 |  |
|  | CT | 151 | 147 | 0.98 (0.69 - 1.40) | 0.91 | 0.95 (0.66 - 1.35) | 0.77 |
|  | TT | 46 | 54 | 0.81 (0.50 - 1.31) | 0.39 | 0.78 (0.48 - 1.27) | 0.32 |
|  | CT+TT | 197 | 201 | 0.93 (0.67 - 1.31) | 0.69 | 0.90 (0.64 - 1.27) | 0.56 |
|  | CC+CT | 257 | 248 | 1 |  | 1 |  |
|  | TT | 46 | 54 | 0.82 (0.54 - 1.26) | 0.37 | 0.81 (0.53 - 1.25) | 0.34 |
| -1985 G>T | GG | 108 | 101 | 1 |  | 1 |  |
|  | GT | 147 | 148 | 0.93 (0.65 - 1.32) | 0.68 | 0.90 (0.63 - 1.28) | 0.55 |
|  | TT | 48 | 53 | 0.85 (0.53 - 1.36) | 0.49 | 0.82 (0.51 - 1.32) | 0.41 |
|  | GT+TT | 195 | 201 | 0.91 (0.65 - 1.27) | 0.57 | 0.88 (0.63 - 1.23) | 0.45 |
|  | GG+GT | 255 | 249 | 1 |  | 1 |  |
|  | TT | 48 | 53 | 0.88 (0.58 - 1.36) | 0.57 | 0.87 (0.57 - 1.34) | 0.53 |
| P1170A C>G | CC | 107 | 100 | 1 |  | 1 |  |
|  | CG | 150 | 148 | 0.95 (0.66 - 1.35) | 0.76 | 0.92 (0.64 - 1.32) | 0.65 |
|  | GG | 46 | 54 | 0.80 (0.49 - 1.29) | 0.35 | 0.78 (0.48 - 1.25) | 0.30 |
|  | CG+GG | 196 | 202 | 0.91 (0.65 - 1.27) | 0.57 | 0.88 (0.63 - 1.24) | 0.46 |
|  | CC+CG | 257 | 248 | 1 |  | 1 |  |
|  | GG | 46 | 54 | 0.82 (0.54 - 1.26) | 0.37 | 0.82 (0.53 - 1.26) | 0.35 |

OR, odds ratio; CI, confidence interval.

**^a^** OR are adjusted for age.

**Additional File 1, Supplementary Table S2- Comparative analysis between genotype frequencies and the risk of lung cancer in the subgroups of smokers**

| Smokers | Genotype | Cases (n=288) | Controls (n=147) | OR (95% CI) | *p*-value | asOR (95% CI) **^b^** | *p*-value |
| --- | --- | --- | --- | --- | --- | --- | --- |
| -3444 C>T | CC | 102 | 49 | 1 |  | 1 |  |
|  | CT | 145 | 77 | 0.91 (0.58 - 1.40) | 0.65 | 0.83 (0.53 - 1.30) | 0.41 |
|  | TT | 41 | 21 | 0.94 (0.50 - 1.76) | 0.84 | 0.93 (0.48 - 1.77) | 0.81 |
|  | CT+TT | 186 | 98 | 0.91 (0.60 - 1.39) | 0.67 | 0.85 (0.55 - 1.31) | 0.45 |
|  | CC+CT | 247 | 126 | 1 |  | 1 |  |
|  | TT | 41 | 21 | 1.00 (0.56 - 1.76) | 0.99 | 1.04 (0.58 - 1.87) | 0.90 |
| -1985 G>T | GG | 103 | 49 | 1 |  | 1 |  |
|  | GT | 142 | 78 | 0.87 (0.56 - 1.34) | 0.52 | 0.78 (0.50 - 1.23) | 0.29 |
|  | TT | 43 | 20 | 1.02 (0.55 - 1.92) | 0.94 | 1.02 (0.53 - 1.95) | 0.96 |
|  | GT+TT | 185 | 98 | 0.90 (0.59 - 1.37) | 0.62 | 0.83 (0.54 - 1.28) | 0.39 |
|  | GG+GT | 245 | 127 | 1 |  | 1 |  |
|  | TT | 43 | 20 | 1.11 (0.63 - 1.98) | 0.71 | 1.18 (0.65 - 2.13) | 0.59 |
| P1170A C>G | CC | 103 | 49 | 1 |  | 1 |  |
|  | CG | 144 | 76 | 0.90 (0.58 - 1.40) | 0.64 | 0.81 (0.52 - 1.28) | 0.37 |
|  | GG | 41 | 22 | 0.89 (0.48 - 1.65) | 0.70 | 0.90 (0.47 - 1.70) | 0.75 |
|  | CG+GG | 185 | 98 | 0.90 (0.59 - 1.37) | 0.62 | 0.83 (0.54 - 1.28) | 0.41 |
|  | CC+CG | 247 | 125 | 1 |  | 1 |  |
|  | GG | 41 | 22 | 0.94 (0.54 - 1.65) | 0.84 | 1.02 (0.57 - 1.82) | 0.96 |

OR, odds ratio; CI, confidence interval.

**^b^** asOR are adjusted for age and gender.

**Additional File 1, Supplementary Table S2- Comparative analysis between genotype frequencies and the risk of lung cancer in the subgroups of drinkers**

| Drinkers | Genotype | Cases (n=192) | Controls (n=146) | OR (95% CI) | *p*-value | asOR (95% CI) **^b^** | *p*-value |
| --- | --- | --- | --- | --- | --- | --- | --- |
| -3444 C>T | CC | 67 | 54 | 1 |  | 1 |  |
|  | CT | 104 | 71 | 1.18 (0.74 - 1.89) | 0.49 | 1.09 (0.67 - 1.79) | 0.73 |
|  | TT | 21 | 21 | 0.81 (0.40 - 1.63) | 0.55 | 0.79 (0.38 - 1.66) | 0.53 |
|  | CT+TT | 125 | 92 | 1.10 (0.70 - 1.71) | 0.69 | 1.02 (0.64 - 1.65) | 0.92 |
|  | CC+CT | 171 | 125 | 1 |  | 1 |  |
|  | TT | 21 | 21 | 0.73 (0.38 - 1.40) | 0.34 | 0.75 (0.38 - 1.48) | 0.41 |
| -1985 G>T | GG | 69 | 54 | 1 |  | 1 |  |
|  | GT | 100 | 72 | 1.09 (0.68 - 1.74) | 0.73 | 0.11 (0.61 - 1.63) | 0.99 |
|  | TT | 23 | 20 | 0.90 (0.45 - 1.81) | 0.77 | 0.90 (0.43 - 1.89) | 0.78 |
|  | GT+TT | 123 | 92 | 1.05 (0.67 - 1.64) | 0.84 | 0.98 (0.61 - 1.57) | 0.92 |
|  | GG+GT | 169 | 126 | 1 |  | 1 |  |
|  | TT | 23 | 20 | 0.86 (0.45 - 1.63) | 0.64 | 0.90 (0.46 - 1.79) | 0.77 |
| P1170A C>G | CC | 68 | 54 | 1 |  | 1 |  |
|  | CG | 104 | 70 | 1.18 (0.74 - 1.89) | 0.49 | 1.10 (0.67 - 1.80) | 0.70 |
|  | GG | 20 | 22 | 0.72 (0.36 - 1.46) | 0.36 | 0.73 (0.35 - 1.54) | 0.41 |
|  | CG+GG | 124 | 92 | 1.07 (0.68 - 1.68) | 0.77 | 1.02 (0.63 - 1.63) | 0.95 |
|  | CC+CG | 172 | 124 | 1 |  | 1 |  |
|  | GG | 20 | 22 | 0.66 (0.34 - 1.25) | 0.20 | 0.69 (0.35 - 1.37) | 0.29 |

OR, odds ratio; CI, confidence interval.

**^b^** asOR are adjusted for age and gender.
